# Multiple Antioxidants Improve Cardiac Complications and Inhibit Cardiac Cell Death in Streptozotocin-Induced Diabetic Rats

**DOI:** 10.1371/journal.pone.0067009

**Published:** 2013-07-02

**Authors:** Santosh Kumar, Sahdeo Prasad, Sandhya L. Sitasawad

**Affiliations:** National Centre for Cell Science, NCCS Complex, Ganeshkhind, Pune, India; Bristol Heart Institute, University of Bristol, United Kingdom

## Abstract

Diabetic cardiomyopathy, a disorder of the heart muscle in diabetic patients, is one of the major causes of heart failure. Since diabetic cardiomyopathy is now known to have a high prevalence in the asymptomatic diabetic patient, prevention at the earliest stage of development by existing molecules would be appropriate in order to prevent the progression of heart failure. In this study, we investigated the protective role of multiple antioxidants (MA), on cardiac dysfunction and cardiac cell apoptosis in streptozotocin (STZ)-induced diabetic rat. Diabetic cardiomyopathy in STZ-treated animals was characterized by declined systolic, diastolic myocardial performance, oxidative stress and apoptosis in cardiac cells. Diabetic rats on supplementation with MA showed decreased oxidative stress evaluated by the content of reduced levels of lipid per-oxidation and decreased activity of catalase with down-regulation of heme-oxygenase-1 mRNA. Supplementation with MA also resulted in a normalized lipid profile and decreased levels of pro-inflammatory transcription factor NF-kappaB as well as cytokines such as TNF-α, IFN-γ, TGF-β, and IL-10. MA was found to decrease the expression of ROS-generating enzymes like xanthine oxidase, monoamine oxidase-A along with 5-Lipoxygenase mRNA and/or protein expression. Further, left ventricular function, measured by a microtip pressure transducer, was re-established as evidenced by increase in ±d*p*/d*t*max, heart rate, decreased blood pressure, systolic and diastolic pressure as well as decrease in the TUNEL positive cardiac cells with increased Bcl-2/Bax ratio. In addition, MA supplementation decreased cell death and activation of NF-kappaB in cardiac H9c2 cells. Based on our results, we conclude that MA supplementation significantly attenuated cardiac dysfunction in diabetic rats; hence MA supplementation may have important clinical implications in terms of prevention and management of diabetic cardiomyopathy.

## Introduction

Cardiomyopathy, a severely disabling complication of diabetes mellitus, is the leading cause of mortality among adults throughout the world [1]. People with cardiomyopathy are often at a risk of suffering from irregular heart beat and sudden cardiac death. The cause of cardiomyopathy is poorly understood. However, oxidative and nitrosative stress induced by reactive oxygen species (ROS) and reactive nitrogen species (RNS) derived from hyperglycemia in the diabetic heart is considered to be a contributing factor in the development and the progression of diabetic cardiomyopathy [2], [3]. The mechanism by which oxidative stress might impair cardiac function involves oxidative damage to cellular proteins and membranes, thereby, inducing cellular dysfunction or death [4]. It has been reported that increased ROS causes cell death through the activation of maladaptive signaling pathways, which could contribute to the pathogenesis of diabetic cardiomyopathy [5]. Whereas, evidence for increased ROS production in diabetes mellitus is reasonably strong [2], [3] the patho-physiological relevance of increased myocardial ROS production in diabetes remains to be further characterized.

Previous studies have demonstrated increased ROS generation from NADPH oxidase [6], [7] and mitochondrial electron transport chain in diabetic myocardium [3], [8] that is thought to play an important role in causing cardiomyocytes apoptosis. However, the mitochondrial enzymatic sources of ROS as well as other non-mitochondrial enzymatic sources of ROS and RNS are less well studied. The outer mitochondrial membrane serotonin-degrading enzymes monoamine oxidases (MAOs) catalyze oxidative deamination of several monoamines (e.g. serotonin [5-hydroxytryptamine], noradrenaline, dopamine), resulting in significant ROS production [9] and recent studies have shown monoamine oxidase A (MAO-A) is an important source of hydrogen peroxide (H_2_O_2_) in the heart [10] known to induce apoptosis in the cardiomyocytes [11]. Further, potential non-mitochondrial sources of ROS could include xanthine oxidase (XO), neuronal nitric oxide synthase (NOS1) and the arachidonic cascade (5-lipoxygenase [5-LO] and cycloxygenase-2 [COX-2]). Although evidence exists for increased production of ROS from NADPH oxidase or reduced neuronal nitric oxide synthase (NOS1) activity coupled with increased activation of XO [12], [13], the role of and possible involvement of other enzymes such as XO, and 5-LO in diabetic cardiomyopathy remain unclear. These enzymes are especially important in redox signaling and may be implicated in the pathophysiology of diabetic cardiomyopathy. In addition, although animal studies with a single antioxidant have shown to produce some beneficial effects in improving cardiac damage [3], [14], [15], studies on high-risk human populations have not produced any beneficial effects [16], [17], suggesting that the relationship between oxidative stress and cardiovascular complications of diabetes is quite complex. Indeed, recent studies suggest that the effects of ROS may be highly dependent upon their source, location, local concentration and perhaps even nature of the ROS species that are generated [18]. In the current study, we have investigated the fate of diabetes on cardiac cells and assessed the protective role of multiple antioxidants (MA) in diabetic cardiomyopathy of animal model. Our results show increased oxidative stress, NF-κB, pro-inflammatory cytokines, dyslipidemia along with increased expression of xanthine oxidase, MAO-A, 5-LO and apoptosis in the diabetic heart and supplementation with MA inhibited these changes.

## Materials and Methods

### Animals and Supplementation

Male Wistar rats (4–6 weeks old) were obtained from the breeding section of the National Centre For Cell Science (Pune, India). Four rats per cage were housed in standard acrylic glass mouse cages in a room maintained at constant temperature and humidity with a 12-hour light:dark cycle; rats were fed regular sterilized chow diet with water *ad libitum*. Our experimental protocol was reviewed and approved by the Institutional Animal Care and Use Committee of the National Centre For Cell Science.

Diabetes was induced in 24 rats by intraperitoneal injection of streptozotocin (STZ, Sigma Chemicals, MO, USA) at the dose of 55 mg/kg dissolved in 0.1 M citrate buffer pH 4.5. Twelve remaining animals were treated with vehicle and were referred to as the control group. After 3 days of STZ injection, the blood glucose levels were measured by using a glucometer (AccuCheck; Roche, Germany). Rats, which had blood sugar values >200 mg/dl, were used for the study. Diabetic rats were randomly segregated into two groups. One group served as diabetic control (STZ), whereas the other was treated with MA in the form of emulsion in drinking water, containing ascorbic acid (200 mg/kg), dl α-tocopherol acetate (50 mg/kg), β-carotene (40 mg/kg), N acetyl cysteine (100 mg/kg), selenium (0.02 mg/kg) and named as STZMA. The selection of the antioxidants was based on previous studies [19]; however the route and doses were modified according to our toxicity studies in vivo. The control groups (*n*  = 12) received either vehicle/water for the same duration. Animals were then supplied with 500 ml water and 30 g food every day. Remaining water volume and food weight was measured 24 h later, and the differences were used to calculate water and food consumption. After induction of diabetes, consumption of food and water was increased in the diabetic rats compared with the controls. Supplementation with MA did not alter the consumption of food and water in diabetic rats. Measurements of glucose and body weight were done every week and at the end of the study. After 12 weeks of supplementation, animals were sacrificed, serum and plasma were separated and tissues were harvested and processed for biochemical measurements.

### Cell Culture and Treatment

H9c2 cells (ATCC CLR-1446; Rockville, MD, USA) were maintained in Dulbecco's modified Eagle's medium (DMEM) supplemented with 10% FBS and 100 units/ml penicillin and 100 mg/ml streptomycin in a humidified atmosphere at 37°C. On reaching 50–60% confluence, the cultures were exposed to high glucose 33 mM as a diabetic condition and low glucose 5.5 mM as a non-diabetic control for 48 hrs [20]. For multiple antioxidants treatments we make mixture of N acetyl cysteine (5 mM), Ascorbic acid (100 µM), α-tocopherol acetate (50 µM), β-carotene (5 µM) and Selenium (100 nM) in a low and high glucose medium and mark as a 1X antioxidants and cell were treated with serial dilution of 1X antioxidants.

### Measurements of Insulin Levels, and Physiological Parameters

Plasma insulin levels were measured by the rat insulin ELISA kit based on the direct sandwich ELISA technique (Mercodia AB, Sweden). Circulating triglycerides, cholesterol LDL, VLDL, HDL, serum glutamic oxaloacetic transaminase (SGOT), serum glutamic pyruvic transaminase (SGPT), uric acid, alkaline phosphatase and Cardiac cTnI levels in rats were measured using a RA -50 semi auto analyzer and HbA1 in analysis was performed by the Nycocard Reader (Ranbaxy, India).

### Left Ventricular Function

At the end of the experiment, rats were anesthetized with urethane (1 g/kg body weight). Then the right carotid artery was cannulated and transducer was advanced into the left ventricle to measure the rate of pressure rise and delay (±dP/dtmax), blood pressure and heart rate with a microtip pressure transducer (SPR-671, Millar Instruments) connected to a Power Lab instrument. Blood pressure and heart rate were monitored and recorded using Chart 5.5 (ADI Instruments, Australia). Rectal temperature was maintained at 37°C throughout the procedure [21].

### Measurement of Oxidative and Nitrosative Stress

Lipid per-oxidation was determined spectrophotometrically on the basis of measurement of chromogen substrate generated from the reaction between malondialdehyde (MDA) and 2-thiobarbiuttric acid (TBA). Catalase assays were done using the kits from sigma (CAT; EC 1.11.1.6). Catalase assay is based on the measurement of the hydrogen peroxide substrate remaining after the action of catalase by colorimetric assay. Heme oxygenase-1 (HO-1) levels in heart tissue were measured by reverse transcriptase PCR. Levels of NO in serum were measured by Griess Reagent Kit (G7921; Invitrogen, USA).

### Measurement of Cytokines in Plasma

TNF-α, TGF-β1, IFN-γ, IL- 4 and IL-10 levels in the plasma were determined by the sandwich ELISA method using a commercially available kit BD OptEIA ELISA Set (BD Biosciences, USA). All appropriate controls and standards as specified by the manufacturer's kit were used and the data are expressed as picogram per milliliter plasma.

### Morphological Analysis and TUNEL Assay

Heart tissue was fixed in 10% formalin, embedded in paraffin, and sectioned at 5-µm thickness for visualization. Sections were stained with hematoxylin and eosin (H&E) and observed under a microscope (Nikon, Eclipse, TE2000-U) with 20× magnification. For TUNEL assay, an *in situ* Cell death detection kit (Roche GmBH, Germany) was used according to the manufacturer’s instructions. Briefly, the slides were deparaffinized and rehydrated using xylene and ethanol gradings and permeablized using hot 0.1 M Citrate buffer pH 6.0 and incubated with the reaction mixture containing TdT and fluorescein labeled dUTP for 1 h at 37°C. Images were captured with a confocal laser scanning microscope (Zeiss LSM510). For negative control, TdT was omitted from the reaction mixture.

### RT-PCR

Messenger RNA was isolated from left ventricular samples. The mRNAs were reverse transcribed into cDNA by using a ThermoscriptTM RT-PCR System (Invitrogen, USA), and the primer pairs listed in Table 1 were used in RT-PCRs to detect XO, MAO-A, HO-1 and actin as a loading control. All reactions were carried out with 1-min denaturation at 94°C, annealing for 1 min at 56°C and elongation at 72°C for 1 min. PCR products were separated by agarose gel electrophoresis [2% (w/v)] and visualized by staining with ethidium bromide.

**Table 1:** Primer sequences for amplification of rat cDNA.

Name of gene	Primer sequence pairs	Annealing Temperature	Product length
HO-1	F: 5″-TcAAcATTgAgcTgTTTgAg-3″R: 5″-AcAggAAacTgAgTgTgAgg-3″	56°C	210
XO	F: 5″-gAcTcAcTTcAAccAgAAgc-3″R:5″-cTggTTcAgAAAAggAAgTg-3″	56°C	193
MAO	F: 5″-TgcATggTgTATTAcAAggA-3″R: 5″-cTTgAgATcccAgAAcTTTg-3″	56°C	238
β-Actin	F: 5″-ccTAgAcTTcgAgcAAgAgA-3″R: 5″-AgAggTcTTTAcggATgTcA-3″	56°C	221

### Western Blot

Heart tissues were rinsed with cold sterile PBS and than homogenized in RIPA Lysis buffer containing protease inhibitor cocktail (Roche Germany). Equal amounts of protein were resolved in SDS-PAGE and transferred to PVDF membrane (Millipore). Blocking was carried out for 1 h in 3% BSA in TBS with Tween 20 (0.1%). The membrane was incubated with primary antibody (XO, 5-LO, NF-κB p65, MAO-A, Bax, Bcl-2, PARP, Nrf-2, GAPDH and Actin) for 3 hour at RT followed by the secondary antibody for 1 hour (Bio-Rad). Protein bands were visualized using the enhanced chemiluminescence substrate reaction (Pierce).

### Preparation of Nuclear Extract from Heart Tissue Samples

Heart tissues (75–100 mg/mouse) from control and treated mice were minced and incubated on ice for 30 minutes in 0.5 mL of ice-cold cytosolic buffer (10 mM HEPES [pH 7.9], 1.5 mM KCl, 10 mM MgCl2, 0.5 mM DTT, 0.1% Igepal CA-630, and 0.5 mM PMSF). The minced tissue was homogenized using a Dounce homogenizer and centrifuged at 16,000×g at 4°C for 10 minutes. The resulting nuclear pellet was suspended in 0.2 mL of nuclear buffer (20 mM HEPES [pH 7.9], 25% glycerol, 1.5 mM MgCl2, 420 mM NaCl, 0.5 mM DTT, 0.2 mM EDTA, 0.5 mM PMSF, and 2 µg/mL leupeptin) and incubated on ice for 2 hours with intermittent mixing. The suspension was then centrifuged at 16,000× g at 4°C for 20 minutes. The supernatant (nuclear extract) was collected and used for NF-κB activation.

### Electrophoretic Mobility Shift Assay

NF-κB activity was determined by electrophoretic mobility shift assay (EMSA) as described elsewhere [22]. In brief, nuclear extract (15 µg) proteins were incubated with ^32^P end-labeled DNA probes for NF-κB (5'AGTT GAGGGGACTTTCCCAGGC3') for 30 minutes at 37°C, and the DNA-protein complex formed was separated from the free oligonucleotide on 6.6% native polyacrylamide gels. The dried gels were visualized with a Storm 820 Phosphor imager, and radioactive bands were quantitated using ImageQuant software (GE Healthcare).

### Analysis of Cell Viability

Cell viability was measured quantitatively by using MTT. Briefly, cells were seeded in 96-well plates (3000 cells per well), and left to adhere to the plates overnight. After exposure to high glucose (33 mM) for 48 hrs, cell viability was assessed by MTT (0.5 mg/ml) conversion as described previously. The absorbance was measured at 570 nm using a Microplate Reader (Molecular Devices, Spectra MAX 250). The data of percentage survival in hypoxia are expressed as the percentage survival of control cell that is culture in Cells were grown at 37°C in a humidified atmosphere of 95% air and 5% CO2.

### Transfection with siRNA

Cells (5×10^5^/well) were plated in 6-well plates and allowed to adhere for 24 hours. Cells were then transfected with 30 nM of p65/RelA-specificor control-siRNAs using oligofectamine (Invitrogen) according to the manufacturer's protocol. Briefly, 2 µL of oligofectamine transfection reagent was added to 100 µL of culture medium, thoroughly mixed, and incubated at room temperature for 15 minutes; 6 µL of siRNA was added to 150 µL of culture medium and then combined with the diluted oligofectamine. The solution was gently mixed, incubated at room temperature for 20 minutes, added to the cell culture, and incubated for the next 48 hours in low and high glucose containing medium. At the end of the incubation, cell lysates were prepared for further analyses.

### Statistical Analysis

Values were represented as mean ± S.E.M. Statistical evaluation of the data was determined by performing the ANOVA followed by Tukey’s post hoc test for multiple comparison. The analysis was performed using the statistical software package (GraphPad-Prism-4, CA, USA) and *P*<0.05 was regarded statistically significant.

## Results

### MA Improved the Diabetic Conditions in Animals

Induction of diabetes in male Wistar rats by STZ led to reduction in the body weight with concomitant decrease in the blood insulin levels and increase in the glucose and HbA1 levels. In addition, STZ increased the cardiac marker TnI in diabetic rats. MA supplementation for 12 weeks significantly improved the insulin levels, body weight and significantly decreased the levels of glucose, and TnI. However, MA did not significantly alter the HbA1 levels in diabetic animals (Table 2).

**Table 2:** Physiological parameters after multiple antioxidants supplementation.

Parameters	Control (n = 6)	STZ * (n = 6)	STZMA ** (n = 6)
Blood Glucose (mg/dl)	130±5.72	576.6±38.28 (P<0.01)	453.0±16.92 (P<0.05)
Blood Insulin (ng/ml)	1.14±0.10	0.30±0.03 (*P*<0.05)	0.50±0.12 (*P*<0.05)
Body weight (g)	376.6±8.43	243.0±4.74 (*P*<0.05)	318.8±7.15 (*P*<0.05)
HbA_1_ (%)	5.25±0.175	9.71±0.31 (P<0.001)	9.3±0.08 (NS)
TnI (ng/ml)	0.95±0.04	1.23±0.42 (P<0.001)	1.01±0.06 (P<0.05)
+Δ*p*/Δ*tmax* (mmHg/s)	4673.69±392.48	1784.01±187.91 (P<0.001)	2839.03±69.22 (P<0.05)
-Δ*p*/Δ*tmax* (mmHg/s)	1921.27±293.23	694.87±18.84 (P<0.05)	645.55±39.61 (NS)
Blood Pressure (mm/Hg)	98.52±1.47	103.66±1.63	100.03±3.04 (NS)
Systolic Pressure (mm/Hg)	118.42±2.35	119.45±1.5250 (P = ns)	117.36±2.49 (NS)
Diastolic Pressure (mm/Hg)	80.10±1.29	88.07±1.86 (P<0.05)	79.93±1.95 (P<0.05)
Heart Rate (Beats/min)	300.8±9.54	221.01±5.63 (*P*<0.001)	256.5±8.88 (*P*<0.0)

* Between Control and STZ, ** Between STZ and STZMA.

### MA Improved the Left Ventricular (LV) Function

Abnormalities of left ventricular function attributed to a "preclinical cardiomyopathy" in diabetic patients. Most parameters for left ventricular function were derived from invasive pressure measurement. We found that diabetic animals have reduced maximal rate of intra ventricular pressure rise [+dp/dtmax (mmHg/s)] and sluggish LV relaxation as indicated by the reduced -dp/dtmax (mmHg/s) compared with control animals, suggesting reduced LV contractility. Supplementation of MA improved the LV contraction in the STZ diabetic group; however no relaxation was observed by MA. Furthermore, diabetic animals showed significant increase in blood pressure and decrease in heart rate. These changes were normalized with MA supplementation (Table 2).

### MA has No Toxic Effects in Diabetic Animals

To determine whether MA induced toxicity in animals, biochemical markers of heart, kidney and liver were investigated. Results showed that uric acid, alkaline phosphatase, and SGPT were increased in diabetic mice (Table 3). Supplementation of MA significantly decreased the SZT induced uric acid and alkaline phosphatase in animals, however no significant change was observed in SGPT level (Table 3). We also found that SGOT, another biomarker of liver and heart, had no significant change either by STZ or MA (Table 3). These results indicate that MA has no cyototoxic effect in animals even it protects the animal’s organ such as heart, liver and kidney.

**Table 3:** Cytotoxic effects of multiple antioxidants supplementation on kidney and liver.

Parameters	Control (n = 6)	STZ * (n = 6)	STZMA ** (n = 6)
Uric acid (mg/dl)	3.68±0.232	4.67±0.167 (*P*<0.05)	4.11±0.11 (*P*<0.05)
Alkaline phosphatase (IU/L)	283.66±13.63	810.0±72.77 (P<0.05)	743.0±55.47 (*P*<0.05)
SGPT (IU/L)	28.50±3.77	61.0±2.86 (*P<0.001)	66.0±5.79 (**NS)
SGOT IU/L)	81.33±5.5780	85.33±5.4810 (*NS)	87.66±8.189 (**NS)

* Between Control and STZ, ** Between STZ and STZMA.

### MA Reduced the Oxidative and Nitrosative Stress

**Figure 1:**
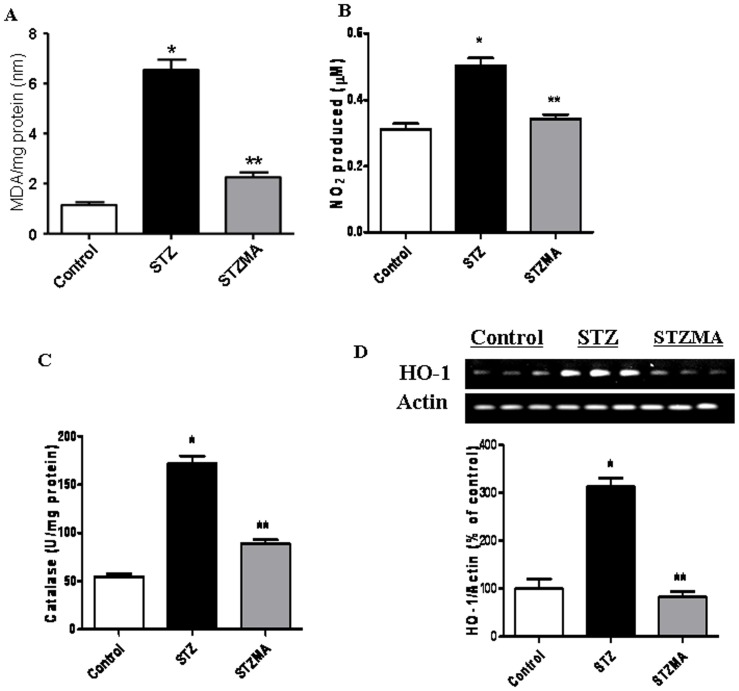
MA decreased the oxidative and nitrosative stress. Effect of MA on (**A**) cardiac tissue lipid peroxides (MDA). *P<0.001 vs. Control group; ** *P*<0.001 vs. STZMA group. (**B**) Serum Nitrite levels. *P<0.001 vs. Control group; ** *P*<0.001 vs. STZMA group. (**C**) Catalase (CAT). *P<0.001 vs. Control group; ** *P*<0.001 vs. STZMA group. (**D**) mRNA expression of hemeoxygenase I (HO-1). Bars demonstrating HO-1 mRNA levels. *P<0.01 vs. Control group; ** *P*<0.001 vs. STZMA group. Results are expressed as means ± SE (*n*  = 6).

Diabetic condition induced the oxidative and nitrosative stress in the myocardial tissues. Our observation also revealed increase in oxidative stress, as indicated by increased lipid peroxidation (nM MDA/mg protein) in the heart tissue of diabetic animal compared with the controls (6.5460±0.4010 vs. 1.1410±0.1180 nM MDA/mg protein, n  = 6, P<0.001) (Fig. 1A). Nitrosative stress detected in the form of nitrite also increased in the serum of diabetic animal compared with that of the controls (0.5030±0.0232 vs. 0.3120±0.0157 mM, n  = 6, P<0.001) (Fig. 1B). Supplementation with MA showed reduction of both lipid peroxidation compared with the diabetic group (STZMA 2.2600±0.1920 nM MDA/mg protein vs. STZ, P<0.001) and NO levels (STZMA 0.3430±0.0122 mM vs. STZ, P<0.001). STZ induced diabetes also showed significant increase in the antioxidant enzymes catalase (Fig. 1C) in cardiac tissue compared with controls (172.2500±7.7170 vs. 54.6170±3.0170 U/mg protein, n  = 6, P<0.001). However, treatment of MA in diabetic animals significantly reduced the catalase levels (STZMA 89.4330±3.4710 U/mg protein vs. STZ, P<0.001). Diabetic group showed remarkable increase in the expression level of HO-1 in cardiac tissue (Fig. 1D) as indicated by increased mRNA level (*P*<0.01). However, diabetic animals supplemented with MA had reduced levels of HO-1 (*P*<0.01). These results thus indicate that oxidative and nitrosative stress induced by hyperglycemia was normalized after multiple antioxidant supplementations.

### MA Decreased the Levels of Pro-inflammatory Cytokines

**Figure 2:**
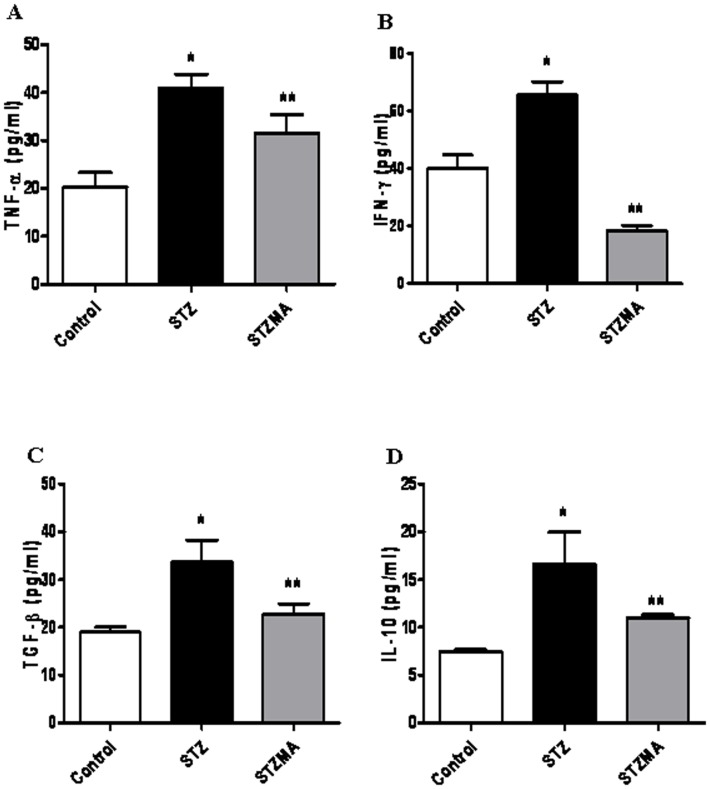
Effects of MA on cytokine levels. (**A**) TNF-α. *P<0.001 vs. Control group;** *P*<0.05 vs. STZMA group. (**B**) IFN-γ. *P<0.05 vs. Control group;** *P*<0.001 vs. STZMA group. (**C**) TGF-β. *P<0.05 vs. Control group;** *P*<0.05 vs. STZMA group. (**D**) IL-10. *P<0.05 vs. Control group;** *P*<0.05 vs. STZMA group in diabetic rats. Results are expressed as means ± SE (*n*  = 6).

Hyperglycemia is known to activate several cytokines by oxidative stress, which might contribute to the development of diabetic complications. Pro-inflammatory cytokine profile revealed significant increase in the plasma cytokine levels in the diabetic animal when compared with the controls. These cytokines include TNF-α (40.9170±2.9200 vs. 20.2670±3.0430 pg/ml, *n  = 6*, *P*<0.001), IFN-γ (65.5500±4.6220 vs. 40.1500±4.5020 pg/ml, *n  = 6*, *P*<0.05), TGF-β (33.6950±4.5540 vs. 18.9690±1.1110 pg/ml, *n  = 6*, *P*<0.05), and IL-10 (16.5670±3.4170 vs. 7.4500±0.2160 pg/ml, *n  = 6*, *P*<0.05). Supplementation with MA distinctly reduced the cytokines IFN-γ (18.5330±1.7100 pg/ml, *n  = 6*, *P*<0.001), TNF-α (31.6000±3.8830 pg/ml, *n*  = 6, *P*<0.05), and TGF-β (22.7440±2.2370 pg/ml, *n  = 6*, *P*<0.05), IL-10 (11.0000±0.3310 pg/ml, *n  = 6*, *P*<0.05). Alteration in the cytokine profiles by multiple antioxidants clearly demonstrates the role of oxidative stress in this process (Fig. 2A–2D).

### MA Supplementation Improves Lipid Profile

**Figure 3:**
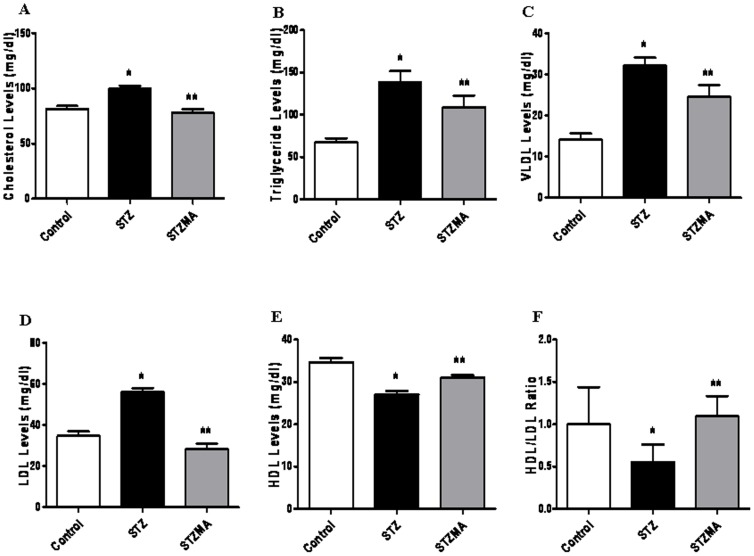
MA improves lipid profile. Effect of MA on serum triglyceride, cholesterol and LDL, VLDL, HDL levels in STZ-induced diabetic rats. Results are expressed as means ± SE (*n*  = 6). (**A**) Cholesterol levels. * *P*<0.05 vs. Control group; **p<0.05 vs. STZMA group. (**B**) Triglyceride levels. *P<0.05 vs. Control group; ** *P*<0.05 vs. STZMA group. (**C**) VLDL levels *P<0.05 vs. Control group;** N.S. vs. STZMA group. (**D**) LDL levels *P<0.05 vs. Control group;** *P*<0.01 vs. STZMA group. (**E**) HDL levels *P<0.05 vs. Control group;**P<0.05 vs. STZMA group. (**F**) HDL/LDL levels *P<0.05 vs. Control group;**P<0.05 vs. STZMA group. Results are expressed as means ± SE (*n*  = 6).

Hyperglycemia-induced oxidative stress is known to alter lipid profile in patients with diabetes. We determined whether multiple antioxidant supplementation modulate serum cholesterol, triglyceride, VLDL, LDL and HDL in diabetic animals. We found that STZ-induced diabetes resulted in markedly increased cholesterol (99.667±2.789 vs. 81.333±2.871 mg/dl, *n  = 6*, *P*<0.05) and triglyceride (138.667±12.837 vs. 67.167±5.056 mg/dl, *n  = 6*, *P*<0.05) levels in the serum compared with the controls. However, animals receiving the MA supplementation (STZMA) significantly lowered cholesterol (81.6670±3.0180, *n  = 6*, *P*<0.05) and triglyceride (109.167±13.688, *n  = 6*, *P*<0.05) levels than the STZ group (Figure 3A-3B). We also observed that STZ-induced diabetic animals had increased levels of VLDL, LDL and decreased levels of HDL in the serum compared with the controls (LDL, 56.0000±1.958 vs. 34.667±2.319 mg/dl, *n  = 6*, *P*<0.05; VLDL, 32.167±1.973 vs. 14.167±1.493 mg/dl, *n  = 6*, *P*<0.05 and HDL, 27.000±0.966 vs. 34.667±1.022 mg/dl, *n  = 6*, *P*<0.05). However, animals receiving MA (STZMA) had significantly lower VLDL (Figure 3C) as well as LDL (Figure 3D) levels and increased HDL levels (Figure 3E) than the STZ diabetic group (LDL, 28.333±2.6160, *n  = 6*, *P*<0.01; VLDL, 24.500±2.930, *n  = 6*, *P*<0.05 and HDL, 31.000±0.632, *n  = 6*, *P*<0.05). MA also improved HDL to LDL ratio in diabetic animals (Figure 3F). These results indicate that MA supplementation significantly reduces hyperlipidemia in diabetes probably through reduction in oxidative stress and thus pro-inflammatory cytokines.

### Regulation of Enzymatic Sources of ROS and Cardiac Apoptosis

Evidences from numerous studies showed that ROS are generated from mitochondrial and non-mitochondrial sources in cardiac tissue [7], [11], [12]. Therefore, we determined whether MA regulates induction of ROS at transcriptional or translational level in the heart tissues. Results of RT-PCR showed up- regulated mRNA expression of XO (*P*<0.05) which produce ROS through NADH oxidation, and MAO-A which in turn produces ROS through oxidative deamination of endogenous and exogenous amines (*P*<0.01) in diabetic rats. However, supplementation of MA inhibited the mRNA expression level of XO and MAO-A significantly.

**Figure 4:**
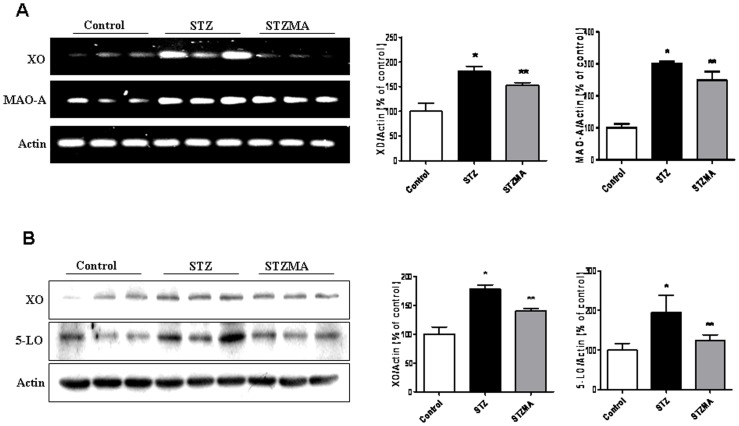
MA regulates enzymatic sources of ROS and cardiac apoptosis. (A) Effects of MA on expression levels of XO, and MAO-A mRNA: mRNA expression of XO. *P<0.05 vs. Control group;** *P*<0.05 vs. STZMA group, MAO-A. *P<0.01 vs. Control group;**NS vs. STZMA group (n = 3) by RT-PCR. (B) Effects of MA on expression levels of XO, and 5-LO protein: protein expression of XO. *P<0.01 vs. Control group;** *P*<0.05 vs. STZMA group, 5-LO. *P<0.05 vs. Control group;**NS vs. STZMA group (n = 3) by western blot.

Further, western blot studies have also showed increased expression of XO (*P*<0.01), and 5-LO (*P*<0.05), indicating ROS are generated in diabetic heart at both transcriptional and translational level. This result also indicates more than one source of ROS in the diabetic heart and hence suggests that these enzymes may contribute to ROS-dependent cardiomyocytes apoptosis. Treatment of MA showed suppressed level of XO and 5-LO suggesting MA inhibit production of ROS in cardiac tissues (Fig. 4).

### MA Inhibits STZ-induced NF-κB Activation in Cardiac Tissue in Mice

**Figure 5:**
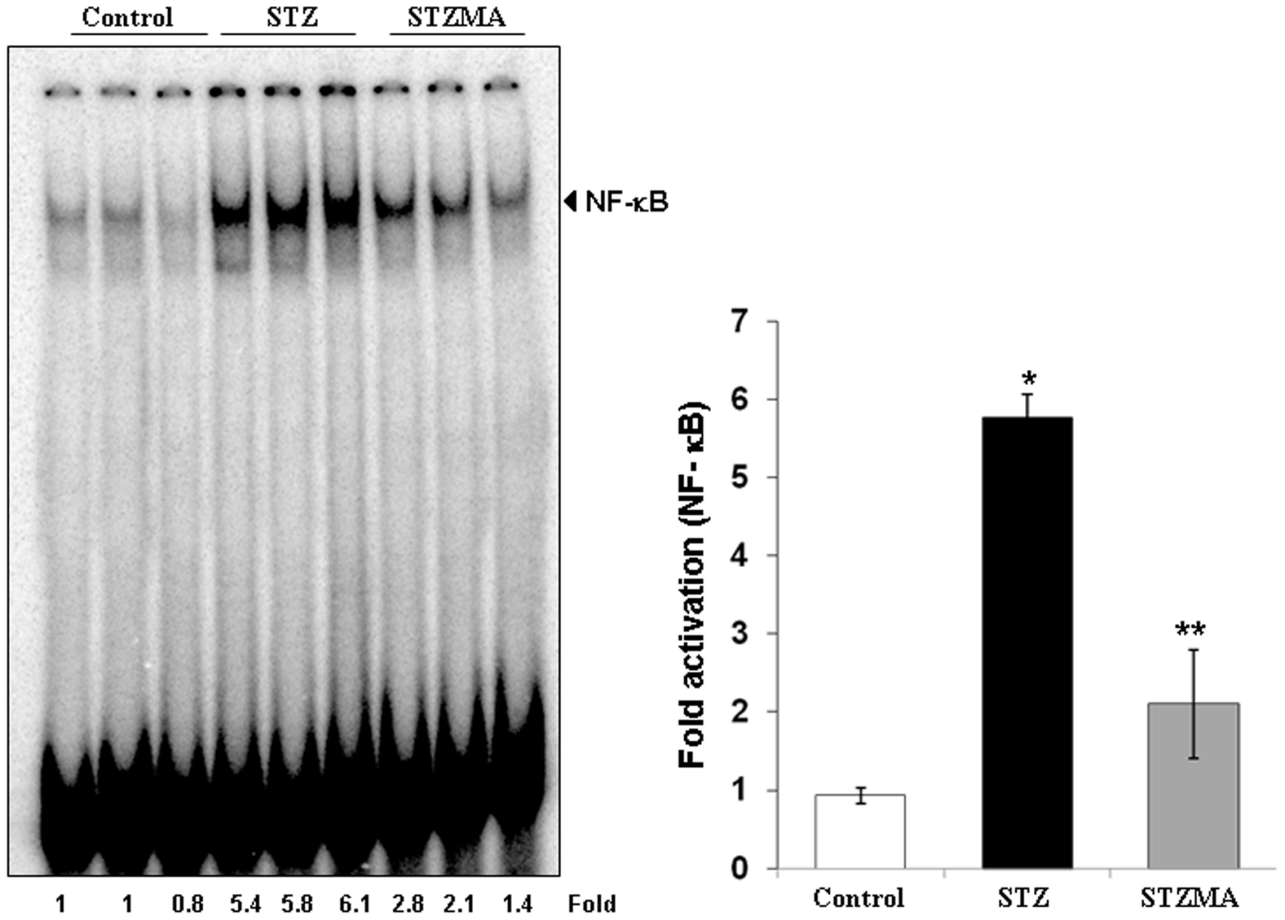
MA inhibits activation of NF-κB in STZ-treated rat cardiac tissues. Nuclear proteins isolated from the heart of treated and control rats were analyzed for NF-κB activation by EMSA as indicated in *Materials and Methods* section. *P<0.001 vs. Control group; ** *P*<0.01 vs. STZMA group (n = 3).

It has been reported that elevated oxidative stress is involved in the activation of the transcription factors NF-κB in the cardiac tissues of diabetic animals, and that this activated NF-κB is associated with the altered gene regulation in the cardiovascular tissues [22]. Therefore, we evaluated whether protective effects of MA against STZ on cardiomyopathy was associated with the inhibition of NF-κB activation. The DNA binding assay for NF-κB in nuclear extracts from treated and untreated animals showed that MA significantly suppressed NF-κB activation in STZ-treated animal’s cardiac tissue (Fig. 5).

### MA Supplementation Inhibits Cardiac Cell Death and Nuclear Translocation of NF-κB

**Figure 6:**
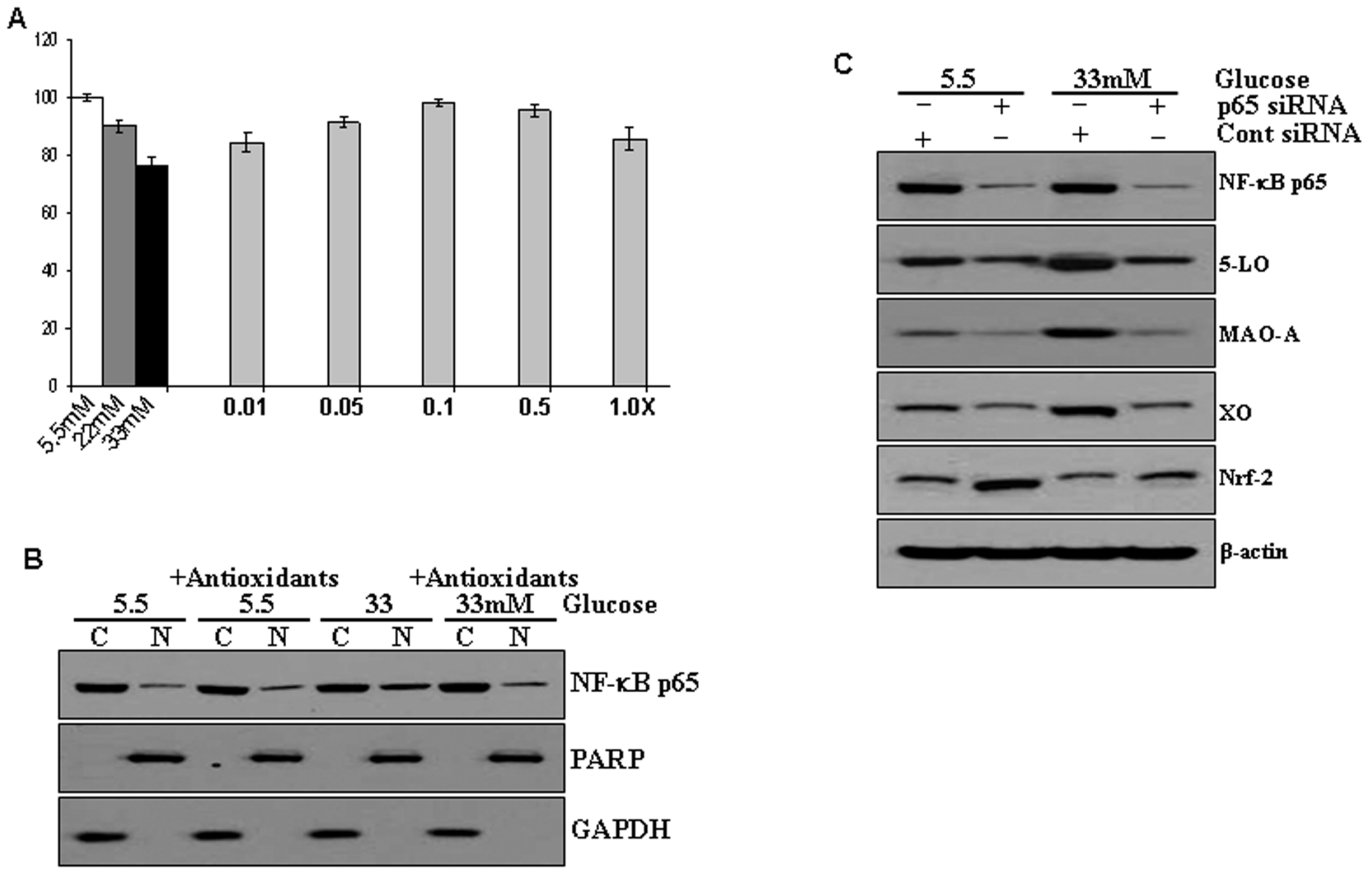
Effects of MA on cardiac cell death and nuclear translocation of NF-κB. (**A**) Cell viability by MTT assay. H9C2 cells were incubated with glucose (5.5, 22, and 33 mM) for 48 hrs. Different concentrations of multiple antioxidant mixture of N acetyl cysteine (5 mM), Ascorbic acid (100 µM), α-tocopherol acetate (50 µM), β-carotene (5 µM) and Selenium (100 nM) (0.01, 0.05, 0.1, 0.5 and 1X) were added for 48 hrs and cell viability was determined by MTT assay; significance of high glucose over control at *P*<0.05. **(B)** Western blot analyses of nuclear and cytosolic fractions prepared from low and high glucose treated H9C2 cells with and without multiple antioxidants mixture (0.1X) for 48 hrs. Membranes were probed with anti-NF-κB p65 antibodies, stripped, and re-probed with GAPDH or anti-PARP antibodies to determine even protein loading and purity of cytosolic [C] and nuclear [N] fractions, respectively. (**C**) Immunoblot analysis of pro-oxidant genes in cells transfected with p65/RelA- siRNA or the vector alone, after transfection H9C2 cells were treat with 5.5 and 33 mM of glucose for 48 hrs. Membranes were probe anti-NF-κB p65, 5-LO, MAO-A, XO and Nrf-2 antibodies. β-actin antibodies to ensure even protein loading in each lane.

We first determined whether glucose induce apoptosis in cardiac H9c2 cells. Our results showed a dose dependent cell death with increasing concentration of glucose (5 mM-33 mM). Further we investigated protective effects of different doses of MA (NAC-5 mM, Ascorbic acid-100 µM, Vitamin E-50 µM, β carotene-5 μ and selenium-100 nM) in glucose-induced cell death. We observed that MA inhibited cell death induced by high concentration of glucose (Figure 6A). Next we determined if high glucose induced the translocation of NF-κB in H9c2 cells. However, we found that MA suppressed the nuclear translocation of p65 NF-κB (Figure 6B). We also investigated the role NF-κB in the expression of 5-LO, MAO and XO. For this, we silenced the p65 NF-κB by using siRNA and determined the expression level of 5-LO, MAO and XO. Results of western blot showed that silencing of p65 NF-κB decreased the expression of these proteins. The findings indicate that NF-κB regulates the expression of 5-LO, MAO and XO, which is associated with growth regulation of cardiac H9c2 cells (Figure 6C). In addition, we found that in diabetic condition Nrf2 expression was decreased in H9c2 cells. However, silencing of p65 resulted in increase of Nrf2 expression level in high glucose exposed as well as control cardiac H9c2 cells (Figure 6C).

### MA Supplementation Inhibits Apoptosis through the Regulation of Mitochondrial Pathway

**Figure 7:**
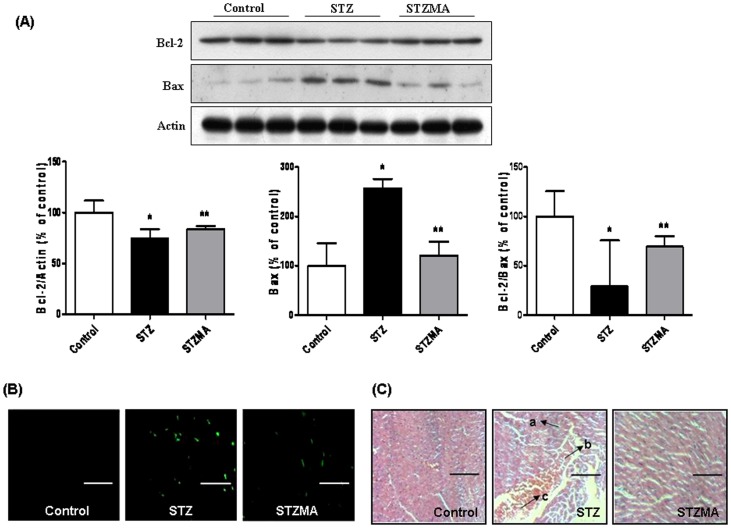
Effects of MA on cardiac cell apoptosis. (**A**) Representative Western blots for Bcl-2, Bax, and actin and bars demonstrating Bcl-2, Bax protein levels, and Bcl-2–to–Bax ratio in control, STZ, and STZMA animals. *Significantly different compared with control and STZ, with *P*<0.05. (**B**) Detection of apoptosis by the TUNEL staining. The green florescent nuclei indicate apoptotic cell death in LV myocardium of control, STZ and STZMA rat, original magnification 20X, scale bar  = 500 µm. (**C**) Representative H&E images of control, STZ and STZMA, which demonstrate marked increased in a) PMN accumulation b) Myocyte fiber diameter and c) Hemorrhagic area (original magnification 20X, scale bar  = 500 µm) in STZ diabetic rat that were prevented after MA supplementation.

Under oxidative stress, mitochondria play an important role in apoptosis and decrease of the Bcl-2/Bax ratio is one of the markers of apoptosis through mitochondrial pathway. Therefore, we further investigated whether MA supplementation also regulates apoptosis. In order to confirm this, we used Bax as a pro-apoptotic and Bcl-2 as an anti-apoptotic marker for apoptosis. Diabetic animals showed elevated cardiac apoptosis as indicated by decreased Bcl-2/actin protein levels (*P*<0.05) and increased Bax/actin levels (*P*<0.01) when compared to control animals. After supplementation with MA, Bcl-2 and Bax levels were comparable to that of control healthy animals and also MA supplementation helps to retain the Bcl-2/Bax ratio compared to control animals (Fig. 7A).

Further the effect of MA on apoptosis in cardiac tissues was determined by TUNEL assay. The extent of cardiac cell apoptosis was low in the control group, however TUNEL-positive cardiomyocytes were dramatically raised in STZ rats (approximately 5-fold vs control) and MA supplementation suppressed this apoptosis (approximately 50% vs STZ, Fig. 7B). Hematoxylin and eosin (H&E) staining of the heart tissues showed that compared with control, diabetic hearts displayed structural abnormalities such as accumulation of polymorphonuclear neutrophils (PMN), degeneration of cardiac myofibrils, a marked separation of myocardial fibers from each other and hemorrhagic areas (Fig. 7C). These structural abnormalities in the heart of STZ diabetic animal were completely prevented by MA supplementation.

## Discussion

The findings of our study indicate that inhibition of oxidative and nitrosative stress with MA improves LV function and minimizes apoptotic cell death in STZ-induced diabetic rat. Besides NADPH oxidase and mitochondrial electron chain as a source of ROS [6], [7], [8], the other enzymatic sources of ROS such as XO, 5-LO and MAO-A also exists in the diabetic myocardium that may play an essential role in diabetic cardiac dysfunction and cardiomyopathy.

Diabetic rats supplemented with MA showed decreased glucose, HbA1 and increased the plasma insulin levels. As β-cells in particular, are highly susceptible to oxidative stress [23], hyperglycemia [24] and dyslipidemia [23], [24] increase in oxidative stress in STZ diabetic rat which may induce apoptosis of β-cell. However multiple antioxidants supplementation in STZ induced diabetes model showed protection of β-cells from further damage by minimizing oxidative stress and dyslipidemia. Although the levels of insulin were increased in the STZMA group compared to STZ group, these were not sufficient to normalize the blood glucose levels.

Accumulating evidence suggests that hyperglycemia induces ROS [15], [25] and overproduction of ROS is associated with apoptosis in the diabetic heart [3], [15]. In response to the increased oxidative stress, the antioxidant enzymes catalase and HO-1, which act as a defense system, are also induced to protect the cell from oxidative stress [14] however; the threshold of protection can vary dramatically as a function of the activity and balance of these enzymes [26]. Consistent with these reports, our results indicated an increase in the levels of lipid peroxidation, nitrite as well as the antioxidants, catalase and HO-1. It is observed that, in case of elevated oxidative stress, cells with increased levels of antioxidants are hypersensitive to oxidative stress rather than protected from it thus rendering the cells resistant to oxidative stress. Thus, an increase in the levels and expression of these antioxidant enzymes in our study may be a compensatory response in the face of elevated oxidative stress. The reason why the increase in HO-1 was accompanied by an increase in catalase is because up-regulation of HO-1 renders the cells resistant to diabetes-induced oxidative stress by increasing other antioxidant genes, including catalase.

Multitudes of experimental and clinical studies have suggested that elevated circulating levels of glucose result in cardiac inflammation and dyslipidemia eventually culminating in cellular dysfunction and death in cardiomyocytes promoting increased remodeling and fibrosis, processes which play a critical role in the development of subsequent diabetic cardiomyopathy [27], [28], [29]. These inflammatory processes are associated with increased oxidative/nitrosative stress. Hyperglycemia activates several cytokines by oxidative mechanisms, which might contribute to the development of diabetic cardiomyopathy [30]. Cytokines attenuate myocyte contractility directly through the reduction of systolic cytosolic calcium via alterations in sarcoplasmic reticulum function and indirectly by down-regulating sarcoplasmic calcium ATPase expression, as reviewed [31]. We found a significant increase in the levels of the pro-inflammatory cytokines TNF-α, IFN-γ, TGF-β1 and IL-10 in the plasma of diabetic group. These levels of cytokines were normalized after MA supplementation. Although increased levels of TGF-β1 may be induced by metabolic abnormalities like hyperinsulinemia and hyperglycemia are implicated in the development of cardiomyopathy [32], [33], [34], the reason for increased levels of IL-10 in our study is not known. Previous studies have shown increased levels of IL-10 in diet-induced obesity in mice [35]. A similar increase in our study could be due to dyslipidemia or oxidative stress. The increase in the levels of cytokines may further activate the enzymes responsible for production of ROS [15], [16], [17], [19].

In addition to hyperglycemia, diabetic patients also commonly suffer from dyslipidemia, characterized in part by defective lipoprotein uptake and metabolism which can lead to increased atherogenesis and incidence of heart disease [36]. To determine if MA supplementation affects lipid metabolism, we examined serum triglyceride, cholesterol, LDL, VLDL and HDL levels. STZ supplementation resulted in markedly elevated serum triglyceride, cholesterol, LDL and VLDL levels and decreased HDL levels compared with control animals, however, supplementation with MA reduced the levels of cholesterol, LDL and VLDL and increased the levels of HDL but did not reduce the levels of triglycerides. The levels of cholesterol and LDL were normalized to those of control animals. Because MA supplementation in diabetic rat improved lipid peroxidation and dyslipidemia in our experimental system, we speculate that hyperglycemia through oxidative stress induced dyslipidemia.

Since substantial evidence suggests the involvement of oxidative stress in the pathophysiology of diabetic cardiomyopathy we further studied the various sources of ROS in the STZ diabetic heart. Although NADPH oxidases and mitochondria are considered to be the primary sources of ROS in the heart, they are also produced by a range of other sources such as xanthine oxidase, monoamine oxidase-A and the arachidonic cascade, and 5-lipoxygenase. The latter enzymes are especially important in redox signaling. Our data shows that intracellular ROS are also produced from different sources in the hearts of STZ diabetic rat. These rat showed increase in the expression of the ROS producing enzymes XO, MAO-A and 5-LO. These results demonstrate the multiplicity of intracellular ROS sources and suggest that ROS generated by different mechanisms and in different locations may have important roles as regulators of cellular metabolism. Our results showed that MA supplementation significantly attenuated the expression levels of XO, MAO-A, and 5-LO. Saraiva et al. [12] showed ROS production increases with increased activation of xanthine oxidoreductase in the myocardium of diabetic mice. Coherent with these findings, an increase in the expression of XO in our study could be due to increased activity of xanthine oxidoreductase. XO reduces NAD**+** leading to the production of both O_2_•− and H_2_O_2_ or XO which catalyzes the oxidation of xanthine and hypoxanthine into uric acid, producing O_2_ •− and H_2_O_2_ as a by-product [37]. Previous studies have shown role of mitochondrial electron transport and NAD(P)H oxidase as a primary sources of ROS in diabetic cardiomyopathy, a major new finding of our study, however, is that there are also another discrete mitochondrial and non-mitochondrial enzymatic source of intracellular ROS that participate in the oxidative burst induced by diabetes in the heart.

The major intracellular target of hyperglycemia and oxidative stress is the transcription factor NF-κB [38], [39], NF-κB, a redox sensitive factor and a key regulator of antioxidant enzymes that can initiate transcription of many genes involved in apoptosis [40]. Hyperglycemia activates several cytokines by oxidative mechanisms, which activates key transcriptional regulators, including NF-κB that might contribute to the development of diabetic cardiomyopathy [30]. Studies have demonstrated that high concentrations of circulating glucose and LDL/VLDL lipoproteins stimulate myocardial NF-κB activation in diabetic cardiomyopathy [41], [42]. NF-κB is also known to activate its downstream inflammatory mediators such as TGF-β_1_, inducible nitric oxide synthase (iNOS), fibronectin, tumor necrosis factor (TNF)-α and interleukin (IL)-1β [43], [44].

We found a marked increase in the binding activity of NF-κB in the heart of 12-week diabetic rats. NF-κB is activated by many factors, including ROS, advanced glycation end products (AGEs), lipopolysaccharide, viral infection, and cytokines [45]. In the present study, treatment with MA reduced the elevated activity of NF-κB in the diabetic hearts, suggesting that the increased oxidative stress in diabetes may also be involved in the activation of NF-κB. In fact, many published data support the possibility of the activation of NF-κB by oxidative stress in vivo [45]. Our finding of an increased lipid peroxide content and nitrite in the heart of diabetic rats also supports this conclusion. Further, the increased activity of NF-κB may result in the alteration of various gene expressions. The binding sites of NF-κB are located upstream of many genes, including those for cytokines [46], growth factors [47] and adhesion molecules [48]. Some in vivo and in vitro data supports the participation of NF-κB in diabetic cardiomyopathy [22]. Therefore, the activation of NF-κB may be related to the altered gene regulation found in the cardiovascular tissues of diabetic subjects. The HO-1 expression and catalase content may be the consequence of these alterations of NF-κB activity because previous studies have shown NF-κB binding sites in the transcriptional regulation of HO-1 expression [49]. Further HO system is known to potentiate antioxidants [50], therefore the decrease in the HO-1 and catalase by MA treatment in our study confirms the association of NF-κB to the increased mRNA content of the enzyme. Also the ability of MA to prevent NO accumulation in our study might be due to the fact that NF-κB–mediated regulation of the inducible form of NO synthase involves ROS [51] or because antioxidants can scavenge NO directly and inhibit formation of cGMP [52].

We demonstrated that reduced cardiac apoptosis could be indicated by normalized Bcl-2/Bax levels due to MA supplementation. Most importantly, study of the other biochemical parameters such as serum uric acid, alkaline phosphatase, SGOT and SGPT levels indicate that an antioxidant regimen used in the present study is not toxic to other organs (Table 3). In our experimental setting although most of the parameters of the cardiac cell dysfunction and death were not normalized, they were prevented significantly. Since oxidative stress has been held responsible for the development of diabetic cardiomyopathy [53] our results show that multiple antioxidants might be helpful in improving cardiac cell apoptosis with consequent contractile dysfunction in diabetic rats. The novelty lies in the fact that this research is the first step in unifying the many factors in the diabetogenic processes in a relevant *in vivo* system and will help move us closer to finding effective treatment or prevention strategies for diabetic cardiomyopathy.
